# Pediatric Diaphyseal Forearm Fracture Management with Biodegradable Poly-L-Lactide-Co-Glycolide (PLGA) Intramedullary Implants: A Longitudinal Study

**DOI:** 10.3390/jcm13144036

**Published:** 2024-07-10

**Authors:** Aba Lőrincz, Ágnes Mária Lengyel, András Kedves, Hermann Nudelman, Gergő Józsa

**Affiliations:** 1 Department of Thermophysiology, Institute for Translational Medicine, Medical School, University of Pécs, 12 Szigeti Street, 7624 Pécs, Hungary; aba.lorincz@gmail.com (A.L.); nuhwaao.pte@tr.pte.hu (H.N.); 2 Institute of Information and Electrical Technology, Faculty of Engineering and Information Technology, University of Pécs, Boszorkány Street 2, 7624 Pécs, Hungary; kedvesandras94@gmail.com; 3 Division of Pediatric Surgery, Traumatology, Urology and Pediatric Otolaryngology, Department of Pediatrics, Clinical Complex, University of Pécs, 7 József Attila Street, 7623 Pécs, Hungary; suzd1j@pte.hu

**Keywords:** pediatric, diaphyseal, forearm, fracture, biodegradable, implants, intramedullary, poly-L-lactide-co-glycolide (PLGA), follow-up

## Abstract

**Background:** Pediatric forearm fractures represent a substantial proportion of childhood injuries, requiring effective and minimally invasive treatments. Our study investigated the mid-term outcomes of biodegradable poly-L-lactide-co-glycolide (PLGA) intramedullary implants in managing diaphyseal forearm fractures in children. **Methods:** A follow-up cohort study was conducted with 38 patients treated with PLGA implants. Control examinations were performed one year post-operation, assessing bone healing through radiographic evaluations and functional outcomes using injured and uninjured limb range of motion (ROM) comparisons. Scarring was evaluated employing the Vancouver Scar Scale (VSS), and satisfaction via a questionnaire. **Results:** Children were predominantly female (76.4%), with a mean age of 9.71 (SD: 2.69) years. Effective fracture stabilization and bone healing were found in all patients, with a minor reduction (mean difference of −1.5°, *p* = 0.282) in elbow flexion on the operated side (139.3°) compared to the intact (140.8°). Elbow extension presented negligible average changes (0.2°, *p* = 0.098). Forearm movements were slightly reduced on the operated side (mean pronation: 80.8° vs. 83.7°, *p* = 0.166; average supination: 83.5° vs. 85.7°, *p* = 0.141). Wrist palmar flexion and dorsiflexion showed no significant differences. VSS ratings indicated minimal scarring (mean guardian and doctor scores were 1.13 and 0.55, respectively, *p* = 0.020), and all patients reported satisfaction with the treatment outcomes. **Conclusions:** Biodegradable implants are effective for pediatric forearm fractures, providing stable bone healing while preserving functional ROM with minimal scarring and high patient satisfaction. PLGA proved to be a viable alternative to traditional metal implants, eliminating secondary removal surgeries.

## 1. Introduction

Among pediatric skeletal injuries, forearm fractures represent a prevalent and clinically significant challenge, accounting for approximately 17.8% of all childhood fractures in the United States, out of an annual incidence rate of 9.47 per 1000 children [1]. The later this damage is adequately addressed, the more severe the resultant loss of forearm motion and subsequent functional and social limitations in performing the activities of daily living, along with the psychological and esthetic impact [2]. The anatomy of the pediatric forearm provides essential guidance for fracture management. Relatively, the ulna is straight and static, while the radius is curved and rotates over the ulna during pronation and supination [3]. These bones are connected by the interosseous membrane in the middle and at the joints—both at the wrist and elbow through the proximal and distal radioulnar joints. Each bone has a proximal and distal physis, with the distal physis contributing significantly to longitudinal growth, accounting for 75% in the radius and 81% in the ulna [4]. Growth polarization correlates with observations that fractures nearer to the distal ends have a higher remodeling potential than those closer to the elbow.

Location and nature of the fracture change with age; complete, displaced fractures are more common in adolescence, while younger children more frequently experience plastic deformation or greenstick fractures [5]. Bilateral forearm fractures often result from indirect trauma, most commonly from falling onto an outstretched hand (FOOSH) [2]. During a fall, a child typically extends the arm to protect against the impact, stiffening the wrist, which leads to fracture due to the axial load that can injure the hand, wrist, forearm, elbow, and shoulder.

Deformities of the limb can indicate a FOOSH injury, but the diagnosis is primarily confirmed through anteroposterior (AP) and lateral orthogonal forearm radiographs, occasionally supplemented by CT or MR scans [6]. Alternatively, a recent trial found the cost-effective, rapid, and radiation-free ultrasound to be a viable diagnostic option [7]. Distal pulses and capillary refill must be assessed too. Direct impacts can also cause isolated ulnar mid-shaft fractures (“nightstick fractures”) or, less commonly, of the radius. Abuse should be considered in children younger than three years old [8].

Classification of pediatric diaphyseal fractures can be performed using the internationally employed “AO Pediatric Comprehensive Classification of Long-Bone Fractures” (PCCF), which provides detailed guidelines for categorizing fractures based on the location and morphology of the break and covering different segments and subsegments of long bones [9]. Fracture location is designated by numbers: the forearm bones are labeled as “2”, the proximal segment as “1”, the diaphyseal as “2”, and the distal as “3”. The letters “r” and “u” denote the radius and ulna, respectively. The specific subsegment of the bone (epiphysis, metaphysis, diaphysis) is indicated by “E”, “M”, and “D”. The second part of the code describes fracture morphology, including patterns specific to children. Severity is separated by a dot (.) and classified into two levels: simple (1) and comminuted (broken into multiple fragments) (2). Displacement is indicated by Roman numerals aiding in a clear and concise description of fractures. In cases where the fracture involves the growth plate, the Salter and Harris classification is utilized [10].

Conservative therapy, which primarily involves immobilization with a cast, is a widely accepted and effective treatment for pediatric forearm fractures, especially when displacement is minimal [8]. Typically, conservative treatment includes closed reduction followed by casting to restore alignment and immobilize the fracture. Casting type and duration depend on the characteristics of the fracture and the age of the child; for example, children under ten with angulations less than ten degrees often achieve complete remodeling and good function with casting alone. Intact periosteum in many of these fractures enhances stability and facilitates natural splinting, making conservative treatment particularly effective. For greenstick fractures, this is especially true, where the bone bends and cracks without breaking completely [11]. Younger children benefit significantly from this method due to the higher remodeling potential of their bones, allowing for healing with minimal intervention and good functional outcomes. However, careful monitoring is essential to prevent complications such as repeated displacement, inadequate healing, or joint stiffness, with follow-up X-rays ensuring proper alignment. Despite these challenges, conservative therapy remains a cornerstone of pediatric fracture management, offering a less invasive option with excellent outcomes in many cases.

Operative therapy serves as a critical intervention for pediatric forearm fractures that are unstable, significantly displaced, involving both the radius and ulna, or unresponsive to conservative treatment [12]. Warranting proper alignment and stability is essential for optimal healing and function preventing long-term complications.

Elastic stable intramedullary nailing (ESIN) is a widely employed minimally invasive procedure for pediatric forearm fractures, which involves inserting flexible, strong metal rods into the medullary cavity of the bone, providing internal stabilization [13,14]. Advantages of ESIN include small incisions, reduced postoperative pain, and fast recovery times while allowing early mobilization, beneficial for maintaining muscle strength and joint function compared to metal plating [12]. Due to some drawbacks of metal implants, new resorbable intramedullary implants have been developed for the surgical treatment of pediatric forearm diaphyseal fractures, composed of biodegradable materials such as poly-L-lactide-co-glycolide (PLGA). They offer temporary support during the healing process while gradually dissolving within the body, eliminating the need for a secondary procedure to remove the hardware and all associated complications [15].

Surgical intervention carries inherent risks, such as nerve injury, infection, or anesthesia-related respiratory depression, but the benefits often outweigh these concerns in appropriately selected cases. Ensuring accurate bone healing, restoring full function, and preventing long-term sequelae such as deformity or chronic pain remain the primary objectives of operative therapy. Therefore, PLGA implants seem particularly advantageous for fractures of the radius, ulna, or both, provided proper immobilization is ensured [16,17]. However, they may not be suitable for oblique spiral, comminuted, or epiphyseal fractures, and are contraindicated in the presence of local infection or poor patient compliance [16].

For complex fractures, plate and screw fixation is often employed to ensure precise alignment of the fracture. This intervention proves particularly effective for fractures near joints or those that are comminuted, providing robust stability and promoting proper healing [8]. External fixation (fixateur externe) is another valuable option involving the stabilization of the bones from outside the body using a frame attached with pins or wires, allowing for adjustment, and it is particularly useful in managing complex fractures with extensive soft tissue injuries [18]. Hybrid fixation combines different methods to achieve optimal results [19].

Despite the apparent advantages of resorbable implants, research on their use in pediatric populations has been limited. Only two studies have investigated the outcomes of intramedullary PLGA implants for pediatric diaphyseal forearm fractures for an extended period, with follow-ups at two [20] and four [17] years post-surgery. Another trial focused on distal fracture management [21]. Therefore, our study aims to assess the mid-term functional and cosmetic results of bioabsorbable intramedullary implants in treating this common condition, providing a deeper understanding of their capabilities in pediatric diaphyseal forearm fractures.

## 2. Materials and Methods

### 2.1. Study Design and Patient Selection

A single-center, single-arm, descriptive cohort follow-up study was conducted at the Pediatric Surgical Division of the University of Pécs in accordance with the Strengthening the Reporting of Observational Studies in Epidemiology (STROBE) guidelines [22]. Data were retrospectively collected from our hospital’s recordings of pediatric patients who consecutively underwent surgery for diaphyseal forearm fractures using PLGA intramedullary implants between May 2021 and March 2023. Then, they were prospectively recalled to evaluate their functional, esthetic, and psychological recovery one year post-surgery.

A total of 38 pediatric patients met the inclusion criteria, which were (1) pediatric patients under the age of 18 years at the time of follow-up with (2) forearm fractures treated with absorbable intramedullary PLGA implants, (3) the time between injury and surgery was within eight days, and (4) they had at least one year of postoperative recovery. Eight patients were excluded due to loss of follow-up attributable to (1) missing contact information, (2) not appearing for control examination, (3) or because of bilateral fractures. Children with (4) oblique spiral, (5) multi-fragmented, or (6) epiphyseal fractures, (7) presence of local infection, (8) poor compliance, and (9) bone remodeling affecting comorbidity or (10) medication would have been also excluded; however, no patient was admitted with these conditions during the investigation period.

### 2.2. Intervention

**Figure 1:**
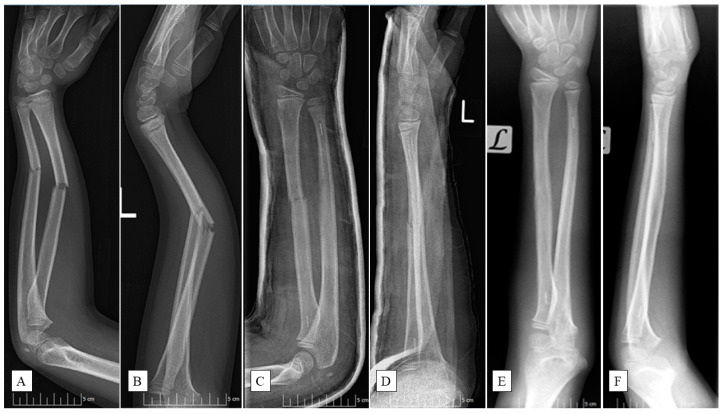
An 11-year-old boy fell while playing football and had a deformed left (L) arm. Preoperative anteroposterior (AP) (**A**) and lateral (**B**) radiographic views of the forearm exposed subperiosteal fractures of both ulna and radius. X-rays post-surgery ((**C**)—AP, (**D**)—lateral aspects) show the successful insertion of PLGA implants, confirmed by the radiopaque markers. Six-month control images ((**E**)—AP, (**F**)—lateral views) revealed completely healed fractures with good axial alignment.

PLGA implants (Activa IM-Nail™, Bioretec Ltd., Tampere, Finland) function by maintaining their mechanical strength throughout the critical period of bone healing (Figure 1).

These polymers degrade through hydrolysis, breaking down into lactic acid and glycolic acid monomers [23,24]. Subsequently, the monomers are metabolized via the citric acid cycle, producing water and carbon dioxide as end products. This process lowers the local pH, creating an acidic environment around the implant which facilitates its gradual resorption. Complete degradation of PLGA typically occurs within 9–12 months, making it a reliable material for temporary internal fixation in pediatric patients [25]. Additionally, the implants feature a tricalcium phosphate (β-TCP) marker for precise fluoroscopic placement (Figure 1C–F).

### 2.3. Surgical Protocol

Several key steps are involved in the surgical procedure for inserting PLGA implants in a minimally invasive manner. After cleaning and disinfecting the operative area and administering general anesthesia, the patient was positioned supine with the affected arm placed on a radiolucent table. Additional management, such as analgesia with 0.1–0.2 mg/kg nalbuphine injections (Nubain, ALTAMEDICS GmbH, Cologne, Germany), and sedation with midazolam (Dormicum, Egis Gyógyszergyár Zrt, Budapest, Hungary), were administered perioperatively based on clinical indications and parental consent. Small incisions were made dorsally on the distal radius and laterally on the proximal ulna. Entry points into the cortical bone had been created using an awl or drill. For the radius, an entry hole was made by positioning the drill perpendicular to the cortex and gradually angling it to form the smallest possible angle with the diaphyseal axis. Then, the medullary canal was reamed with a dilator that matched the implant size—and this process was repeated for the ulna. Implant diameter choice is critical and should match the smallest diameter of the medullary canal, with available options being 2.7 and 3.2 mm in diameter, and lengths of 200, 300, and 400 mm.

Once the canals were prepared, the PLGA implants were introduced using an inserter, guaranteeing no rotational movements to prevent misalignment. Fluoroscopy was used to verify implant positions, with the β-TCP tips aiding in visualization. Protruding ends of the implants were trimmed and smoothed to avoid soft tissue irritation, and the incisions were closed with absorbable sutures. Postoperative care includes immobilizing the limb in a cast above the elbow at a 90-degree angle for 4–6 weeks, with sports and strenuous activities avoided for 2–6 months.

### 2.4. Evaluated Metrics

**Figure 2:**
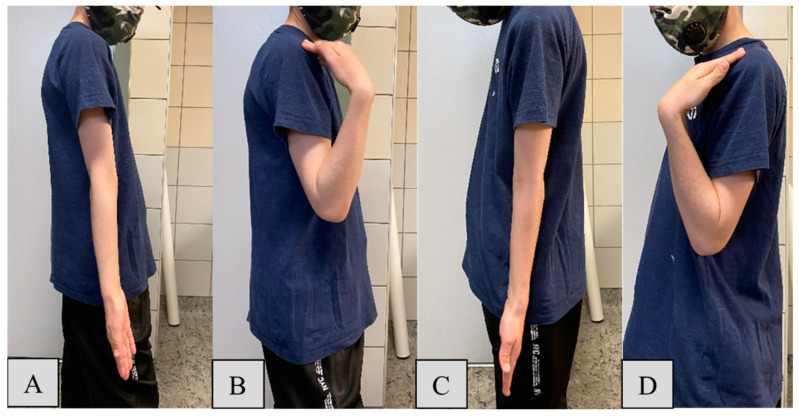
Elbow extension (**A**) and flexion (**B**) of the uninjured arm of an 11-year-old boy. One year after surgery, the operated arm’s cubital extension (**C**) and flexion (**D**) demonstrate intact functionality.

**Figure 3:**
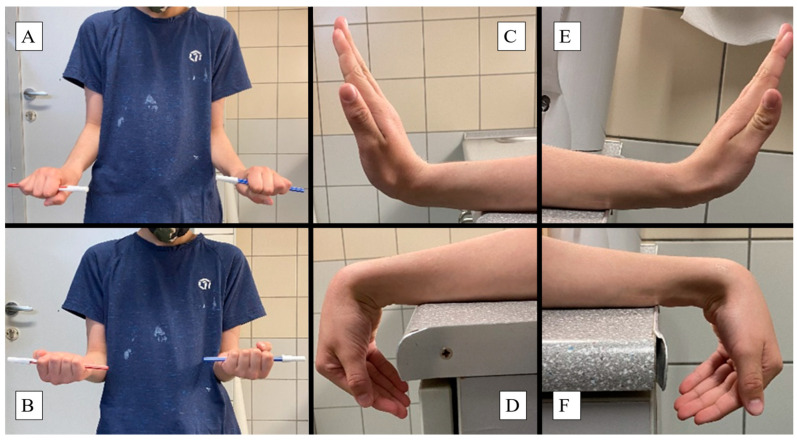
One year post-PLGA implantation: Images showing the pronation (**A**) and supination (**B**) of the forearm, while the intact hand holds a red pen and the healed grasps the blue writing implement. Baseline wrist function is demonstrated with the dorsal (**C**) and palmar flexion (**D**) of the uninjured extremity, which can be compared to the ROM of the operated arm (**E**,**F**).

Endpoints included patient demographics (such as age, sex, dominant hand) and fracture characteristics (time of injury, and affected side and bone), collected in Microsoft Excel 2021 (Microsoft Corporation, Redmond, WA, USA). Primary outcomes included the joint function evaluation one year after surgery via their range of motion (ROM) using a goniometer. ROM was calculated by adding the absolute values of opposing motions. Measurements from the operated extremity were compared to those of the unharmed limb to determine any discrepancies in ROM for the following movements:

- Elbow flexion and extension: Normal range is −10 to 150 degrees (°). Patients were instructed to fully extend and flex their elbows while standing with arms at their sides (Figure 2).
- Forearm pronation and supination: The standard interval is 80 to 90° in both directions from the neutral position. Children held a pen in a fist with elbows at 90°, rotating their forearms to achieve maximum pronation and supination (Figure 3A,B).
- Wrist palmar flexion and dorsiflexion: Typical ROM is 80° dorsiflexion and 70° palmar flexion. Patients placed their forearms on a horizontal surface, moving their wrists to the maximum palmar and dorsiflexion positions (Figure 3C–F).

**Figure 4:**
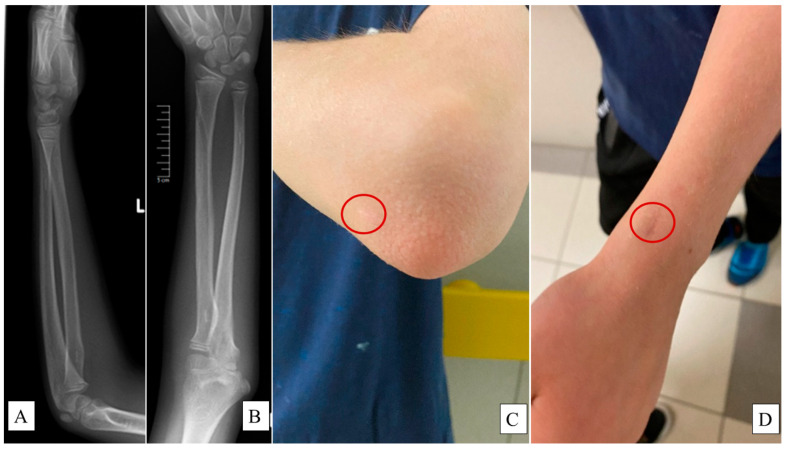
X-rays captured at the one-year follow-up examination of a L pediatric forearm fracture treated with PLGA implants from lateral (**A**) and AP (**B**) views showing intact bone growth and development. Surgical scars—highlighted by the red circles—were evaluated via the Vancouver Scar Scale (VSS) on the olecranon ((**C**), 0 point) and the distal end of the radius ((**D**), 1 point).

X-rays were employed to assess the remodeling of the bones (Figure 4A,B), while surgical scars were evaluated using the Vancouver Scar Scale (VSS) (Figure 4C,D) [26], calculating the composite score of four criteria:

- Pigmentation: scored from 0 (normal) to 2 (severe hyperpigmentation).
- Vascularity: counted from 0 (normal) to 3 (severe vascularity).
- Pliability: graded from 0 (normal) to 5 (severe contracture).
- Height: recorded from 0 (flat) to 3 (more than 5 mm).

Satisfaction was assessed through a questionnaire asking if the patient or their guardian would choose the same surgical method again under similar circumstances. This survey aimed to capture subjective satisfaction with the functional and aesthetic outcomes of the surgery.

### 2.5. Data Analysis and Visualization

Descriptive statistics were calculated until two decimals for all continuous outcomes utilizing means, standard deviations (SDs), medians, interquartile ranges (IQRs), 25th percentiles (IQR25s), 75th percentiles (IQR75s), counts, and ranges, while discrete endpoints were analyzed via count and percentage distributions. This study employed Python 3.12.3 (Python Software Foundation, Wilmington, DE, USA) for data visualization and statistical analysis, a versatile and open-source language that facilitated data handling and testing operating several specialized libraries. Statistical analysis was conducted using SciPy and NumPy libraries. The Shapiro–Wilk test was utilized to determine normality, suitable for smaller sample sizes. Two-sample *t*-tests were used to compare means from two independent groups when both samples were normally distributed, while nonparametric Mann–Whitney U-tests differentiated not normally distributed samples. Additionally, a chi-square (χ^2^) test determined the association between categorical variables. Differences were deemed significant at *p* ≤ 0.05. Matplotlib was utilized for fundamental plot creation and customization, while Seaborn provided advanced plotting functions and aesthetic enhancements.

## 3. Results

This study included 38 pediatric patients with diaphyseal forearm fractures treated using PLGA implants. Patients age ranged from 5 to 15 years, with a mean age of 9.71 (SD = 2.69) years. Regarding sex distribution, the majority of the patients (76.32% of all cases) were female, and the affected side was more often the right forearm (55.26%, *n* = 21) (Table 1). Dominant hand analysis revealed that 85.71% (*n* = 18) of the patients were right-handed; moreover, the nondominant hand was involved in slightly more (52.12%, *n* = 12) injuries, which was not statistically significant (*p* = 0.513). In terms of fracture type, most of the fractures involved both the radius and ulna (84.21%, *n* = 32), with only five children (13.16%) having fractures of the radius alone and one patient (2.63%) having a fracture of the ulna alone. Restricted elbow flexion (<137°) was linked significantly (*p* = 0.017) with radius-only fractures (80% of patients had limited mobility), while wide ROM (≥137°) was marginally associated (*p* = 0.052) with fractures of both bones (64.52% of children had high mobility).

**Figure 5:**
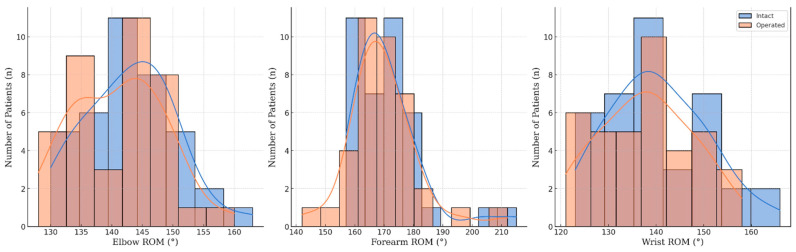
Range of motion (ROM) distributions of pediatric forearm fractured patients one year after PLGA implantation.

Functional outcomes were assessed via ROM for various movements of the forearm, including the elbow and wrist performance as well, and are summarized in Table 2 and visualized in Figure 5.

For elbow flexion, the mean maximum angle for the operated side was 139.3° (SD = 6.2), compared to 140.8° (SD = 6.2) on the intact side. The minor reduction of −1.45° on the operated side suggests effective restoration of function by the PLGA implants, despite a statistically significant difference (*p* = 0.282). Elbow extension showed mean ROM values of −1.1° (SD = 2.9) for the PLGA-treated and −1.3° (SD = 2.9) for the intact side, with a negligible mean difference of 0.05° (*p* = 0.098), indicating that the surgical intervention had no significant impact on extension capability.

Forearm pronation exhibited a mean ROM of 80.8° (SD = 6.6) on the operated side and 82.4° (SD = 6.6) on the intact side. The slight reduction of −1.61° in pronation on the operated side (*p* = 0.166), suggests a clinically minimal impact on rotational movement. Supination of the forearm demonstrated a mean ROM loss of 0.24° (*p* = 0.141) for the operated side, representing no significant difference and, thus, supporting the efficacy of the PLGA implants in maintaining rotational movement.

Wrist dorsiflexion showed a mean difference of −0.34° (*p* = 0.070) between the operated and intact sides, representing no significant impact on wrist extension and underscoring the implant’s capacity to maintain wrist flexibility. Palmar flexion measurements indicated a mean difference of −0.89° (*p* = 0.563) between the operated and intact sides, showing no statistically significant variation and highlighting the implant’s ability to preserve a wide range of wrist movements.

For the total scores, guardians rated the forearm scars with a mean score of 1.13 (SD = 1.14), while medical professionals provided a significantly (*p* = 0.020) lower mean VSS score of 0.55 (SD = 0.80). Patient satisfaction was universally high, with all 38 patients reported as satisfied with the treatment outcomes. Comparisons are presented in brief in Table 3.

## 4. Discussion

Restoring function while ensuring proper bone healing is the primary objective of pediatric fracture management [8]. Our results indicate that PLGA intramedullary implants are effective in achieving this balance. PLGA is a biodegradable polymer that has garnered attention for its biocompatibility, controlled degradation properties, and minimal toxicity. Due to these characteristics, it is also one of the most promising drug delivery systems in nanoparticle formulation [27]. It is possible to add further active ingredients (such as IGF1) into the implant with timed-release properties, for osteostimulation or to prevent infections [28]. In pediatric traumatology, its features are particularly advantageous, as the implants gradually degrade, eliminating the need for a second surgery to remove hardware [15]. Fewer interventions reduce the overall healthcare burden and mitigate the psychological impact of additional surgical interventions on young patients and their families. Eliminating a second surgical procedure also translates into fewer anesthesia-related risks and a reduced likelihood of peri- and postoperative complications, such as nerve injuries, bleeding, infections, or scar tissue formation, which are particularly pertinent in pediatric populations [29]. Furthermore, the shorter hospital stays and reduced need for follow-up visits significantly decrease the disruption to a child’s education and social life, which are vital for their overall development. Despite PLGA materials being generally well tolerated, the potential for allergic reactions or adverse responses in certain patients should be investigated. Additionally, PLGA implants are nearly invisible on X-ray imaging, while they are compatible with MRI. To decrease the cost of control examinations and better compliance, β-TCP bits were incorporated into the tips of the implants, which show up as hyper-opacities on X-ray.

Expanding on our previous investigations [16,30], the current cohort included 38, predominantly female (76.32%), pediatric patients, generally with both of their diaphyses (84.21%) fractured on a single forearm with a mean age of 9.71. A novel observation was that originally, slightly limited elbow flexion (<137°) correlated significantly (*p* = 0.017) with radius-only fractures (80% of patients had restricted mobility), while a broad original ROM (≥137°) was marginally associated (*p* = 0.052) with fractures of both diaphyseal bones (64.52% of children had high mobility)—which might also be significant within a larger analyzed population.

A minor reduction in mean elbow (absolute flexion difference: −1.45°, *p* = 0.282; extension: 0.05°, *p* = 0.098), forearm (pronation: −1.61°, *p* = 0.166; supination: 0.24°, *p* = 0.141), and wrist (palmar flexion: −0.89°, *p* = 0.563; dorsiflexion: −1.55°, *p* = 0.070) mobility highlights the precision of these implants in preserving near-normal ROM. A randomized controlled trial (RCT) found similar patterns regarding preserved ROM using PLGA intramedullary implants [20]. Moreover, they showed that the current gold standard ESIN utilization slightly reduced forearm rotational ROM on the injured side, and increased postoperative pain compared to PLGA intramedullary implants. These findings are significant given the crucial role of elbow and forearm movements in daily activities and play, which are essential for children’s development and quality of life. Consistent functional outcomes across ages, genders, and different fracture types underscore the versatility and reliability of PLGA implants.

Another study found that they were also applicable in osteochondral fractures of the lateral condyle of the femur, patella, and radial head [31]. Therefore, the observed uniformity suggests that surgeons can confidently employ these implants across a wide range of fractures, ensuring optimal outcomes irrespective of specific characteristics. This adaptability could be further enhanced by individualizing management to patient needs, considering factors such as the dominant hand and specific activity requirements. Advances in 3D printing and bioengineering could enable the creation of such patient-specific PLGA implants. Tailoring the size and shape of the implants to fit the unique anatomical and physiological needs of each child may improve the effectiveness and comfort of the treatment.

Our results also indicate a generally positive outcome for pediatric forearm fractures treated with PLGA implants, with high satisfaction rates and minor VSS scores, suggesting minimal scarring as assessed by both guardians and doctors. Although both groups gave low total VSS scores, it must be emphasized that guardians rated the scars 104.76% worse than healthcare professionals, which correlates with recent observations [32]. Educating patients and their families about the benefits and care associated with PLGA implants can enhance compliance and satisfaction. Clear communication regarding the implant’s degradation process and expected recovery timeline is essential for setting realistic expectations.

Traditional methods of managing pediatric forearm fractures often involve metallic implants, which, while effective, necessitate removal surgeries and pose risks of long-term complications such as hardware irritation or migration [15]. A rigid, nondegradable material may increase postoperative pain and distress, which can lead to a less comfortable recovery period for pediatric patients. Corrosion or mechanical wear can exacerbate cellular toxicity and tissue reactions [33]. Chronic inflammation due to metal debris may also play a role in carcinogenesis [34]. Using PLGA implants circumvents these issues, offering a more patient-friendly approach with fewer long-term risks. Given that PLGA degrades over time, it might also affect the growth plate less [23,24]. A disadvantage of the degradation process is that it is theorized to lead to intermediary byproducts, which are acidic in nature and halt osteoblast activity, thereby hindering recanalization. This has been studied in maxillofacial surgeries and pediatric pelvic osteotomies with over 90% bone recanalization within two years, and another investigation regarding the bone regrowth of the implant canal is underway by Hedelin et al. [35,36]. On the other hand, one of its byproducts, lactate, has a crucial part in biochemical pathways and could exert therapeutic effects such as angiogenesis [37]. Another important consideration is the environmental impact of materials. Metal implants contribute to medical waste and require energy-intensive production processes [38]. In contrast, PLGA implants degrade naturally within the body, reducing the ecological footprint of surgical interventions, and aligning with the broader global efforts toward sustainability in healthcare practices.

Still, there may be cases that require additional stability, such as morbidly obese or hyperactive, noncompliant patients because inadequate fixation can potentially lead to structural rotation, displacement, and shifting. Children with complex, open, pathological, or previous fractures, significant soft-tissue injury, infection in the forearm, metabolic bone or systemic disease, medication affecting bone quality, or fractures older than fourteen days were not yet treated with PLGA and reported in the literature; therefore, careful consideration is necessary [16,17,20]. According to an animal study, resorbable PLGA implants only maintained their maximum stability for eight weeks; thus, if prolonged bone healing or extreme mechanical stress is expected, metallic implants might be more appropriate—albeit they were not analyzed again until the six-month postoperative follow-up, where significant implant resorption was observed [25]. In addition to its accessibility and safe conservation of alignment, ESIN is easier to remove than other operative methods following placement. It also requires shorter periods of hospital admission and anesthesia compared to open approaches [39,40]. ESINs are clearly visible on radiographs and, thus, are easier to manipulate than resorbable implants; however, they are incompatible with magnetic imaging. According to a modified Clavien–Dindo Classification, ESIN was connected to a 9% chance of grade 1 (i.e., asymptomatic delayed union—particularly in children older than 10 years) and 17% of grade 2–4 surgical complications [41]. When using PLGA, there were two implant failures (10.5% of the examined population) [17] and one refracture (1.3%) reported in the literature [16], which were 2.5% (*n* = 5) for ESIN in both cases [41]. However, we did not encounter these or any complications during the investigation—in addition to the aforementioned slight ROM reductions and minor scars.

Future perspectives and limitations of this research must also be discussed. Expanding the study to include larger and more diverse populations whose data are collected prospectively in a randomized and blinded manner will provide more comprehensive insights into the generalizability of these findings and reduce possible bias. While the current study corroborates the efficacy of PLGA implants in maintaining functional outcomes, future research should explore the long-term effects of these implants on bone health and growth. Given the dynamic nature of pediatric bone development, it is essential to monitor how the gradual degradation of PLGA implants influences bone remodeling over more extended periods. Longitudinal studies tracking patients into adolescence and adulthood would provide valuable insights into any delayed effects and further solidify the implants’ safety profile. Additionally, investigating the economic impact of PLGA implants, including cost-effectiveness analyses directly compared to traditional methods, could further substantiate their adoption in clinical practice. Potential research could explore their usefulness in other pediatric traumas, such as fractures of the femur, tibia, or even more complex, multi-fragmentary fractures. Expanding the use of PLGA implants could standardize treatment protocols and streamline surgical training for pediatric surgeons. Lastly, the scar scales and questionnaires are subjective measurements, which could be further objectivized by specialized instruments, such as laser Doppler imaging (LDI) for quantifying scar blood flow, Cutometers for measuring elasticity, or ColorMeters for calculating the melanin index [42]. Using advanced imaging techniques, for example, MRI, would also reveal implant resorption rates [17].

Management of pediatric forearm fractures using PLGA intramedullary implants has demonstrated promising outcomes, reflecting advancements in biomaterial technology and surgical techniques. Our study presents compelling evidence supporting the capabilities of PLGA implants in maintaining functional ROM while minimizing complications, thereby enhancing the overall recovery experience for young patients.

## 5. Conclusions

Our study demonstrates that PLGA intramedullary implants effectively restore function and ensure proper bone healing in pediatric diaphyseal forearm fractures. Children showed only a minor reduction in mobility, indicating the precision of these implants in preserving a near-normal ROM. Consistent functional outcomes across different ages, genders, and fracture types highlight the versatility of PLGA implants. They offer a patient-friendly alternative to traditional metallic implants, which often require removal surgeries and pose long-term risks such as hardware irritation or migration. The biodegradable nature of PLGA eliminates the need for a second surgery, reducing healthcare burdens and psychological impacts on young patients and their families while decreasing anesthesia-related risks and peri- and postoperative complications such as vessel or nerve injuries, infections, or scar tissue formation. Additionally, fewer hospital stays and follow-up visits minimize disruptions to a child’s education and social life, crucial for their development. While PLGA’s degradation process may introduce some intermediary byproducts, the overall benefits seem to outweigh these concerns.

Future research should focus on the long-term effects of PLGA implants on bone health and growth, expanding to larger, diverse populations to validate findings. Additionally, investigating the economic impact and exploring their application in other pediatric orthopedic conditions could further substantiate PLGA implants’ adoption in clinical practice. Currently, PLGA implants represent a significant advancement in pediatric fracture management, enhancing recovery experiences for young patients.

## Figures and Tables

**Table 1:** Discrete outcome distribution of children and their PLGA-treated diaphyseal forearm fractures, one year post-operation.

Variable	Category	Count	Percentage
Sex	Female	29	76.32%
	Male	9	23.68%
Affected side	Left	17	44.74%
	Right	21	55.26%
Fracture type	Radius	5	13.16%
	Both	32	84.21%
	Ulna	1	2.63%
Dominant hand	Right	18	85.71%
	Left	3	14.29%
Satisfaction	Satisfied	38	100.00%

**Table 2:** Descriptive statistics of continuous variables from 38 PLGA-treated, diaphyseal forearm fractured children, based on data collected during one-year follow-up examinations.

Endpoint (Unit)	Region	Status	Mean	SD	Min	Max	Median	IQR	IQR25	IQR75
Age (years)	∞	Child	9.71	2.69	5.00	15.00	10.00	4.00	8.00	12.00
Flexion (°)	Elbow	Operated	139.30	6.20	130.00	155.00	140.50	8.25	136.50	144.75
		Intact	140.80	6.20	130.00	155.00	140.50	8.25	136.50	144.75
Extension (°)		Operated	−1.10	2.90	−10.00	0.00	0.00	3.00	−3.00	0.00
		Intact	−1.30	2.90	−10.00	0.00	0.00	3.00	−3.00	0.00
Pronation (°)	Forearm	Operated	80.80	6.60	70.00	90.00	85.00	6.75	83.25	90.00
		Intact	83.70	6.60	75.00	90.00	85.00	6.75	83.25	90.00
Supination (°)		Operated	83.50	6.60	72.00	110.00	84.00	5.00	80.00	85.00
		Intact	85.70	6.60	75.00	90.00	85.00	6.75	83.25	90.00
Palmar Flexion (°)	Wrist	Operated	64.90	6.70	50.00	75.00	68.00	5.75	65.00	70.75
		Intact	68.60	6.10	50.00	80.00	70.00	7.00	66.50	73.50
Dorsiflexion (°)		Operated	73.20	6.70	60.00	86.00	68.00	6.00	65.00	71.00
		Intact	74.20	6.40	64.00	86.00	72.00	5.00	70.00	75.00
VSS (total score)	Upper Limb	Guardian	1.13	1.14	0.00	4.00	1.00	2.00	0.00	2.00
		Doctor	0.55	0.80	0.00	3.00	0.00	1.00	0.00	1.00

**Table 3:** Comparative endpoints one year postoperatively in children with diaphyseal forearm fractures treated with PLGA.

Measurement	Absolute Difference	Relative Difference	Test Used	*p*-Value
Elbow Flexion (°)	−1.45	1.03%	Two-sample t	0.282
Elbow Extension (°)	0.05	3.70%	Two-sample t	0.098
Pronation (°)	−1.61	1.31%	Mann–Whitney U	0.166
Supination (°)	0.24	−3.55%	Mann–Whitney U	0.141
Palmar Flexion (°)	−0.89	1.31%	Two-sample t	0.563
Dorsiflexion (°)	−1.55	−3.55%	Mann–Whitney U	0.070
Dominant Hand Fracture (*n*)	−1	−4.74%	Chi (χ^2^)-squared	0.513
VSS (Total Score)	0.58	104.76%	Mann–Whitney U	0.020

## Data Availability

All data are contained within the article.
